# Nuciferine-Loaded Lipid Nanoparticle Microneedles Alleviate Intervertebral Disc Degeneration by Restoring Nucleus Pulposus Cell Homeostasis

**DOI:** 10.3390/pharmaceutics18080997

**Published:** 2026-08-13

**Authors:** Yifan Ding, Genchun Wang, Yucheng Wang, Songwei Tan, Yixin Cai

**Affiliations:** 1 Department of Thoracic Surgery, Tongji Hospital, Tongji Medical College, Huazhong University of Science and Technology, Wuhan 430030, China; dingyifan@tjh.tjmu.edu.cn (Y.D.); wangyucheng01@hust.edu.cn (Y.W.); 2 Department of Joint Surgery, Orthopedic Medical Center, Fujian Medical University Union Hospital, Fuzhou 350001, China; wgcdoc@fjmu.edu.cn; 3 School of Pharmacy, Tongji Medical College, Huazhong University of Science and Technology, Wuhan 430030, China

**Keywords:** intervertebral disc degeneration, nuciferine, lipid nanoparticles, microneedles

## Abstract

**Background/Objectives**: Intervertebral disc degeneration (IDD) is a major cause of chronic low back pain and disability. Nuciferine, a natural alkaloid with mitochondrial protective activity, shows therapeutic potential for IDD. However, the avascular nature of intervertebral discs limits effective drug delivery. This study aimed to develop a hierarchical stimuli-responsive microneedle (MN) system for localized nuciferine delivery. **Methods**: Nuciferine-loaded lipid nanoparticles (LNPs) were embedded in a phenylboronic acid-modified hyaluronic acid (HA-PBA) matrix to construct functional MNs. LNP characteristics and drug release were evaluated under physiological and simulated degenerative conditions. The effects of released LNPs on nucleus pulposus cell (NPC) viability, mitophagy, oxidative stress, and extracellular matrix homeostasis were assessed in vitro. Therapeutic efficacy was further evaluated in a rat IDD model. **Results**: Nuciferine-loaded LNPs had a mean size of 86.54 nm, PDI of 0.108, and encapsulation efficiency of 75.65%. The HA-PBA MNs showed minimal drug release at pH 7.4 but markedly accelerated release under dual stimuli (100 μM H_2_O_2_ and pH 5.0). Released LNPs maintained NPC viability above 90%, enhanced mitophagy by increasing LC3B-II and decreasing P62, and alleviated oxidative damage and extracellular matrix imbalance. In vivo, MN treatment significantly preserved the disc height index and improved Pfirrmann grades compared with the IDD group (*p* < 0.05). **Conclusions**: The hierarchical stimuli-responsive MN system enables localized, pathological microenvironment-responsive nuciferine delivery and protects against IDD by promoting mitophagy, reducing oxidative damage, and restoring extracellular matrix homeostasis. This strategy provides a potential approach for localized treatment of IDD.

## 1. Introduction

Intervertebral disc degeneration (IDD) is one of the most common musculoskeletal disorders worldwide and represents the leading cause of low back pain, which imposes enormous social and economic burdens [1]. At the molecular level, IDD is driven by a complex interplay of pathological processes, including progressive extracellular matrix (ECM) degradation mediated by upregulated matrix metalloproteinases (MMPs) and ADAMTS enzymes, accumulation of reactive oxygen species (ROS) that overwhelm cellular antioxidant defenses, mitochondrial dysfunction and impaired mitophagy in nucleus pulposus cells (NPCs), sustained pro-inflammatory cytokine signaling (TNF-α, IL-1β, IL-6), cellular senescence accompanied by a senescence-associated secretory phenotype (SASP), NPC apoptosis, and progressive impairment of nutrient and oxygen diffusion resulting from endplate calcifications [2,3]. These interconnected mechanisms collectively disrupt disc homeostasis and drive the progressive structural deterioration characteristic of IDD. Current therapeutic approaches, including conservative management such as analgesics and physical therapy, and surgical interventions such as discectomy or spinal fusion, primarily address symptoms rather than the underlying pathological mechanisms [4]. Although intradiscal injections of biologics or drugs have shown promise for disease modification, their invasive nature and potential complications limit their widespread application [5]. To overcome these limitations, a variety of emerging local delivery strategies have been explored for IDD treatment, including injectable hydrogels that form drug depots within the disc space [6], exosome-based therapies leveraging paracrine signaling [7,8], mesenchymal stem cell delivery for tissue regeneration, gene therapy approaches for durable molecular correction, and conventional intradiscal drug injections [9]. However, each of these modalities carries inherent drawbacks—injectable systems require direct intradiscal access with associated infection risk, exosome and stem cell therapies face production scalability and immunogenicity challenges, and gene therapy raises unresolved safety and regulatory concerns. The emergence of microneedle (MN)-based drug delivery systems offers a new avenue for minimally invasive, localized, and controlled drug administration to the disc space, attracting increasing attention as an emerging treatment modality for IDD [10]. Notably, dissolvable and stimuli-responsive MN systems possess several properties that make them particularly well-suited for IDD therapy: they painlessly penetrate the skin barrier without hypodermic needles, enable spatial targeting of the disc through the overlying skin, support on-demand drug release triggered by the pathological disc microenvironment, and eliminate the complication risks associated with intradiscal puncture. The scalable fabrication and patient-friendly transdermal application of MN patches further enhance their translational appeal [11].

Mitophagy, the selective autophagic clearance of damaged mitochondria, serves as a critical intracellular quality control mechanism [12]. In nucleus pulposus cells (NPCs), mitophagy is essential for maintaining mitochondrial homeostasis. During IDD, autophagic flux in NPCs is frequently impaired, leading to the accumulation of dysfunctional mitochondria that continuously generate reactive oxygen species (ROS) and trigger inflammatory cascades [13]. The excessive production of ROS and the secretion of pro-inflammatory cytokines mutually reinforce each other, forming a self-perpetuating “oxidative stress–inflammation” vicious cycle [14,15]. This cycle drives the degradation of the extracellular matrix (ECM) and progressive destruction of disc structure. At the cellular level, this pathological milieu simultaneously activates apoptotic cascades (via the Bax/Bcl-2 axis), promotes p21/p53-dependent cellular senescence and SASP, suppresses anabolic ECM synthesis (collagen II, aggrecan), and accelerates catabolic ECM breakdown through MMP and ADAMTS upregulation, ultimately culminating in irreversible NPC dysfunction and disc structural failure [16]. Accordingly, restoration or enhancement of mitophagy to break this pathological loop has emerged as a highly promising therapeutic intervention in IDD.

Natural Chinese medicines play a vital role in the treatment of musculoskeletal disorders [17]. As shown in Figure S1, nuciferine (Nuc), an aporphine alkaloid derived from lotus leaves (*Nelumbo nucifera*), has recently emerged as a bioactive compound with multiple pharmacological activities, including anti-inflammatory [18], antioxidant [19], and mitophagy-inducing properties [20]. Mechanistically, nuciferine promotes PINK1 stabilization on depolarized mitochondria and facilitates Parkin recruitment and ubiquitination of outer mitochondrial membrane proteins, thereby targeting damaged mitochondria for selective autophagic clearance via the PINK1/Parkin pathway [21]. This mitophagic activation restores mitochondrial membrane potential, reduces ROS generation from dysfunctional electron transport chains, and suppresses downstream NF-κB-mediated inflammatory signaling [22]. Existing studies have confirmed that nuciferine protects against high-fat-diet-induced hepatic steatosis and insulin resistance by activating the mTORC1/TFEB-dependent autophagy pathway [23]. However, its application in the context of IDD remains unexplored. Given the hydrophobic nature of nuciferine and the challenging microenvironment of the disc, efficient drug delivery represents a critical bottleneck. Lipid nanoparticles (LNPs) are clinically validated nanocarriers composed of four functional components: an ionizable lipid (e.g., DLin-MC3-DMA) that enables pH-dependent endosomal escape, a helper phospholipid (DSPC) that provides bilayer structural integrity, cholesterol that regulates membrane fluidity and stability, and a PEGylated lipid (DMG-PEG2000) that confers steric stabilization and prolonged colloidal stability [24]. Compared with alternative nanocarrier systems—including polymeric nanoparticles, liposomes, and inorganic nanoparticles—LNPs offer superior biocompatibility derived from endogenous lipid components, highly efficient intracellular trafficking via endosomal escape, sustained hydrophobic drug release profiles, and robust protection of labile payloads against enzymatic degradation [25]. LNPs have been successfully employed for the delivery of hydrophobic alkaloids and polyphenolic compounds structurally analogous to nuciferine, demonstrating their versatility as carriers for this class of bioactive molecules [26,27]. Lipid nanoparticles (LNPs) are well-established delivery vehicles with excellent biocompatibility and encapsulation efficiency for hydrophobic compounds, making them ideal carriers for nuciferine [28,29,30].

**Scheme 1:**
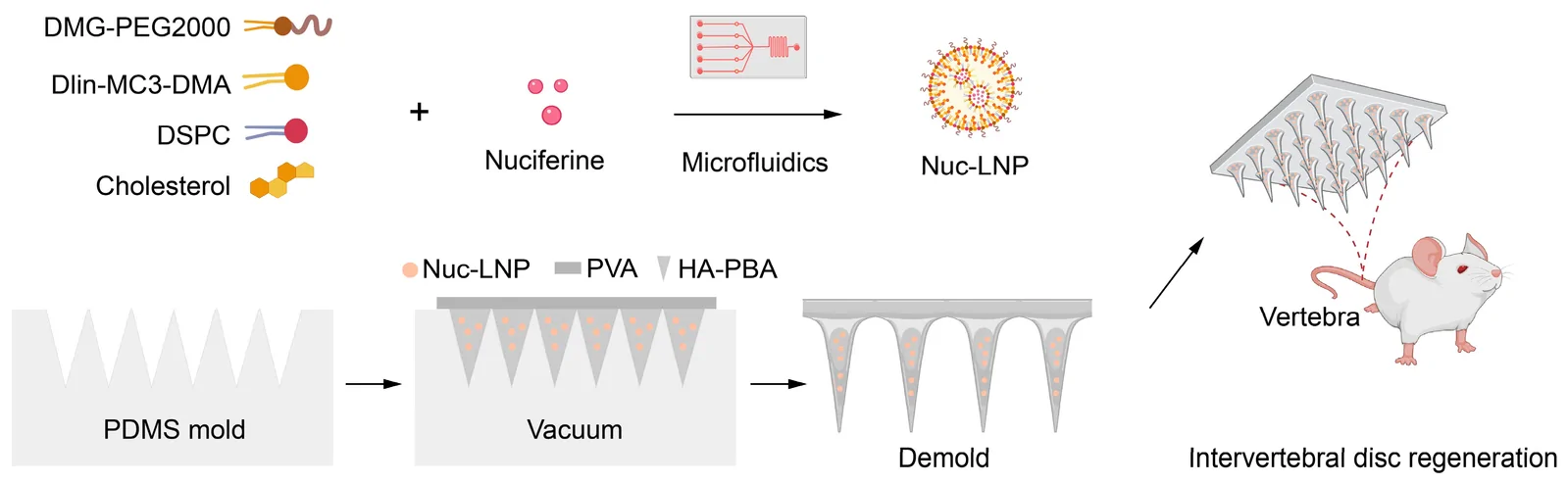
Preparation of Nuc-LNPs and Nuc-LNP-loaded microneedles for intervertebral disc regeneration.

In this study, we designed a nuciferine-loaded lipid nanoparticle microneedle system (Nuc-LNP@MN) to achieve minimally invasive, microenvironment-responsive delivery for IDD treatment (Scheme 1). The microneedle matrix was fabricated from phenylboronic acid-grafted hyaluronic acid (HA-PBA), which is capable of dual ROS/pH-responsive drug release exploiting the elevated ROS levels and acidic environment characteristic of degenerated discs [31,32,33]. The boronic ester bonds within HA-PBA are cleaved under high ROS concentrations, while the pH-sensitive protonation of PBA facilitates dissolution under acidic conditions, collectively enabling on-demand drug release [34]. Released Nuc-LNPs are then taken up by NPCs to activate mitophagy, clear damaged mitochondria, and ultimately break the oxidative stress–inflammation vicious cycle. The efficacy of this strategy was validated in vitro using IL-1β-stimulated NPCs and in vivo in a rat needle-puncture IDD model [35]. To the best of our knowledge, this is the first report of a nuciferine-loaded LNP microneedle platform that integrates ROS/pH dual-responsiveness with mitophagy-targeted therapy for IDD, representing a multi-level innovation that distinguishes this approach from previously reported single-modality delivery strategies. This work provides a novel, stimulus-responsive nanomedicine strategy for mitophagy-targeted IDD therapy.

## 2. Materials and Methods

### 2.1. Materials

HA-PBA (hyaluronic acid, MW 150 kDa; phenylboronic acid substitution degree 25%) and PVA (MW 75 kDa) were purchased from Engineering For Life (EFL Company, Suzhou, China; Product No. EFL-HA-PBA001). MC3 (DLin-MC3-DMA), DSPC (1,2-distearoyl-sn-glycero-3-phosphocholine), cholesterol, and DMG-PEG2000 were obtained from AVT Pharmaceutical (Shanghai, China). Nuciferine (purity ≥ 98%) was purchased from MedChemExpress (HY-N0049; MedChemExpress, Monmouth Junction, NJ, USA). The Calcein-AM/PI live/dead staining kit, CCK-8 assay kit, and DCFH-DA ROS detection kit were obtained from Servicebio (Wuhan, China). Anti-P62, anti-LC3B, anti-Col2a1, and anti-GAPDH antibodies were obtained from Abclonal (Abclonal Technology, Wuhan, China). All other reagents were of analytical grade unless otherwise stated.

### 2.2. Preparation of Nuciferine-Loaded Lipid Nanoparticles (Nuc-LNPs)

Nuc-LNPs were prepared using a microfluidic mixing approach [36]. Briefly, nuciferine (2 mg) and lipid components [Dlin-MC3-DMA 50 mol%, DSPC 10 mol%, cholesterol 38.5 mol%, DMG-PEG2000 1.5 mol%] were dissolved in anhydrous ethanol to prepare a lipid phase at a total lipid concentration of 12.5 mM. The aqueous phase consisted of 50 mM citrate buffer (pH 4.0). The two phases were rapidly mixed through a herringbone-patterned microfluidic chip at a volumetric flow rate ratio of 3:1 (aqueous phase to lipid phase) with a total flow rate of 8 mL/min. The resulting mixture was collected and transferred into a 20K MWCO dialysis bag (Spectrum Laboratories, Rancho Dominguez, CA, USA), followed by dialysis against PBS for 16 h to remove ethanol and achieve solvent exchange, yielding Nuc-LNPs. Plain LNPs (without nuciferine) were prepared identically as a control. The hydrodynamic particle size and PDI of Nuc-LNPs were measured by dynamic light scattering (DLS) mode, and the zeta potential was detected by electrophoretic light scattering (ELS) mode based on the laser Doppler electrophoresis principle. Both assays were carried out on a ZetaPlus analyzer (Brookhaven Instruments, Holtsville, NY, USA). Before detection, the Nuc-LNP suspension was diluted with ultrapure water to a suitable concentration to eliminate multiple scattering interference. All measurements were performed at room temperature, and each sample was tested in three parallel replicates.

To determine encapsulation efficiency and drug loading, a standard calibration curve for nuciferine was established. Briefly, 2.2 mg nuciferine was dissolved in 880 μL anhydrous ethanol, followed by the addition of 3.52 mL ultrapure water to obtain a stock solution of 500 μg/mL in 25% ethanol. Serial two-fold dilutions were performed using 25% ethanol to generate standard solutions ranging from 3.9 to 62.5 μg/mL. Absorbance was measured at 270 nm (the maximum UV absorption wavelength of nuciferine) using a UV spectrophotometer. The encapsulation efficiency (EE%) was calculated as EE% = (total drug − free drug)/total drug × 100%.

### 2.3. Fabrication and Characterization of Nuc-LNP@MN

Microneedle arrays were fabricated using a vacuum-assisted molding method with polydimethylsiloxane (PDMS) molds [37]. The needle tip material was prepared by dissolving HA-PBA in ultrapure water at a concentration of 5% (*w*/*v*), followed by the addition of Nuc-LNPs. The Nuc-LNP-loaded HA-PBA solution was pipetted onto the PDMS mold and subjected to vacuum (approximately −60 kPa) for 3–4 cycles (3 min per cycle) to fill the needle cavities completely. Excess solution was removed, and the mold was air-dried at room temperature for 12 h. Subsequently, a 20% (*w*/*v*) PVA solution was cast onto the mold surface to form the backing substrate layer and dried at 37 °C for an additional 24 h. The resulting microneedle patches (Nuc-LNP@MN) were carefully peeled from the molds and stored at room temperature until use. Blank microneedle arrays (MN) and nuciferine-only microneedles (Nuc-MN) were prepared as controls using the same method.

The morphology of the microneedle array was first captured using a smartphone (Mate 60 Pro, Huawei, Shenzhen, China) to obtain macroscopic images of the overall patch and needle tips. Microscopic morphology was further characterized by scanning electron microscopy (SEM, Zeiss Sigma 300, Oberkochen, Germany) after gold sputter-coating. The mechanical properties of the microneedle arrays were evaluated using a universal testing machine (Instron 5943, Instron, Norwood, MA, USA). Briefly, individual microneedle patches were placed on a flat steel plate with needles pointing upward, and a flat-ended cylindrical probe descended at a constant rate of 0.1 mm/min until a predetermined displacement was reached. The compression force–displacement curves were recorded to assess the failure force and stiffness of the microneedles. To evaluate skin penetration capability, the microneedle patches were pressed onto freshly excised porcine skin for 1 min under a constant force of approximately 20 N. The puncture performance was assessed by staining the skin surface with trypan blue solution, followed by visualization and quantification of the puncture holes.

### 2.4. In Vitro Drug Release Study

The in vitro drug release profile of Nuc-LNP@MN was first investigated to demonstrate the stimulus-responsive release behavior of the HA-PBA matrix. The boronic ester bonds of HA-PBA undergo oxidative cleavage under elevated ROS conditions and hydrolytic cleavage under acidic conditions; therefore, the release experiment was performed using a Franz diffusion cell system under different simulated microenvironments. As reported in previous research [38], specifically speaking, Franz diffusion cells were utilized in this study to evaluate the transdermal delivery performance of microneedles (MNs), with 25% aqueous ethanol adopted as the receiving solution. Freshly excised abdominal rat skin was used as the experimental membrane; hair was removed and subcutaneous fat was trimmed off to simulate the transdermal delivery pathway of microneedles. MNs loaded with nuciferine–lipid nanoparticles (nuciferine–LNPs) were applied onto isolated rat skin with the stratum corneum facing upward. The skin was pressed for 1 min to pierce the epidermal layer before the device was fixed. Vertical Franz diffusion cells (PermeGear, Hellertown, PA, USA) were employed for the experiment, which possessed a receiving chamber volume of 12 mL and an effective diffusion area of 1.77 cm^2^. A magnetic stir bar was placed inside the receiving chamber, and the stirring speed was set to 400 rpm, with the whole transdermal penetration experiment maintained at a constant temperature of 37 °C. To assess the stimulus-responsive release behavior, the receptor media were tailored to simulate the IDD microenvironment, including: PBS (control, pH 7.4), PBS containing 100 μM H_2_O_2_ (ROS-mimicking environment), PBS adjusted to pH 5.0 (acidic environment), and a combination of both conditions. At predetermined time intervals (0.5, 1, 2, 4, 6, 8, 12, 24, and 48 h), the concentration of nuciferine in the collected aliquots was quantified via UV spectrophotometry (Agilent Technologies, Santa Clara, CA, USA) at 270 nm, and the cumulative drug release percentage was subsequently calculated.

### 2.5. Isolation and Culture of NPCs

As previously reported [4], intact disc tissues were carefully separated from Sprague-Dawley rats weighing between 200 and 250 g. The extracted NP tissues were diced and primarily digested at 37 °C for approximately 45 min using a 0.25% trypsin solution supplemented with 0.01% EDTA (Biothink Biological Technology, Nanjing, China). A secondary enzymatic dissociation was then performed using 0.25% collagenase type II (Gibco, Grand Island, NY, USA) at 37 °C for 3 h. The released NPCs were then seeded and expanded in DMEM/F12 supplemented with 10% FBS (Gibco) and 1% antibiotic mixture (Biothink Biological Technology). The culture environment was maintained at 37 °C with 5% CO_2_ and standard oxygen levels (21% O_2_). Cells from passages 0 through 2, which displayed no discernible phenotypic changes compared to primary cells, were selected for downstream evaluations.

### 2.6. Cytotoxicity Assessment

The cytocompatibility of Nuc-LNPs and the Nuc-LNP@MN system was evaluated using both the CCK-8 assay and Calcein-AM/PI live/dead labeling. As described in our previous published work [5], in the CCK-8 assay, NPCs were seeded in 96-well plates at 5 × 10^4^ cells per well and incubated overnight. Cells were then exposed to graded concentrations of Nuc-LNPs (0.2, 0.8, 4, 8, and 12 µM nuciferine equivalent) for 24 h. After adding CCK-8 reagent and incubating for 2 h, absorbance at 450 nm was measured using a microplate reader. To mimic a clinically relevant Transwell co-culture system, Nuc-LNP@MN patches were positioned in the upper chamber inserts (0.4 µm pore size), whereas NPCs were seeded in the lower wells, evaluating indirect cytotoxicity. For live/dead labeling, cells were stained with Calcein-AM (2 µM) and PI (4 µM) over 30 min at 37 °C, followed by fluorescence imaging using a fluorescence microscope.

### 2.7. Immunofluorescence Staining

To evaluate the effect of Nuc-LNP@MN treatment on collagen II (Col2a1) expression in IL-1β-stimulated NPCs, immunofluorescence staining was performed. Briefly, NPCs were seeded on glass coverslips, pre-treated with IL-1β (10 ng/mL) for 24 h, and then treated with the respective groups. Following treatment, cells underwent fixation with 4% paraformaldehyde for 15 min, permeabilization with 0.1% Triton X-100 for 10 min, and blocking with 5% bovine serum albumin (BSA) in PBS over 1 h. Cells were then incubated with an anti-Col2a1 primary antibody (1:200 dilution) overnight at 4 °C. Following washing, cells were exposed to an Alexa Fluor 594-conjugated secondary antibody (1:500) for 1 h at room temperature. F-actin was labeled with FITC-conjugated phalloidin, and nuclei were counterstained using DAPI. Fluorescence images were acquired using a confocal laser scanning microscope (CLSM).

### 2.8. Western Blot Analysis

Protein levels of mitophagy markers (P62 and LC3B) were assessed by Western blot. NPCs were lysed in RIPA buffer containing protease and phosphatase inhibitors on ice for 30 min. As reported in our previous publications [39], protein concentrations were quantified with a BCA assay. Equal amounts of protein (30 µg) were separated by SDS-PAGE on 10–12% gels and transferred to PVDF membranes. Membranes were blocked with 5% skimmed milk in TBST for 1 h, and then probed with primary antibodies against P62 (1:1000), LC3B (1:1000), and GAPDH (1:5000) overnight at 4 °C. After washing with TBST, HRP-conjugated secondary antibodies were applied for 1 h at room temperature. Protein bands were developed using an ECL chemiluminescence detection kit (Servicebio Technology Co., Ltd., Wuhan, China), and band intensities were quantified using ImageJ software (v 1.53). To confirm the role of autophagy in the therapeutic mechanism, 3-methyladenine (3-MA, 5 mM), an autophagy inhibitor, was added 1 h prior to Nuc-LNP@MN treatment in specific experimental groups.

### 2.9. Quantitative RT-PCR

Total RNA was isolated from NPCs with TRIzol reagent (Takara Bio, Kusatsu, Japan). RNA concentration and purity were assessed using a NanoDrop spectrophotometer (Thermo Fisher Scientific, Wilmington, DE, USA). Complementary DNA (cDNA) was reverse-transcribed from 1 µg of total RNA using the PrimeScript RT reagent kit (Nanjing Vazyme Biotech Co., Ltd., Nanjing, China, Cat. # R223). Quantitative real-time PCR was carried out using SYBR Green PCR Master Mix (Nanjing Vazyme, Cat. # Q711) on a real-time PCR platform. GAPDH served as the internal reference, and the relative transcript levels of target genes including MMP3 and MMP13 were computed via the 2^−ΔΔCt^ approach. Primer sequences applied in this study are provided in Table 1.

### 2.10. ROS Detection

Intracellular ROS was quantified with the DCFH-DA fluorescent probe (Servicebio, Wuhan, China). After the corresponding treatments, NPCs were washed with serum-free medium and incubated with 10 µM DCFH-DA at 37 °C for 30 min. Cells were subsequently washed three times in PBS, and the fluorescence signal of 2,7-dichlorofluorescein (DCF) was recorded with a fluorescence microscope. The average fluorescence intensity was quantified as an indicator of intracellular ROS levels.

### 2.11. Animal Model and In Vivo Experiments

All animal experiments were approved by the Fujian Medical University Experimental Animal Ethics Committee. Male Sprague-Dawley (SD) rats (7–8-week-old) were used to establish the needle-puncture IDD model. Under general anesthesia, the coccygeal intervertebral discs (Co8/Co9 and Co9/Co10) were located under palpation guidance. A 21-gauge needle was inserted into the nucleus pulposus and rotated 360° to induce disc degeneration, with Co7/Co8 serving as the normal control group, Co8/Co9 as the IDD model group, and Co9/Co10 as the IDD plus treatment group. At 2 weeks after puncture, microneedle patches were applied to the skin overlying the intervertebral discs and maintained for 20 min. The Co9/Co10 discs were treated with different formulations (Nuc, Nuc-LNPs, or Nuc-LNP@MN; nuciferine equivalent dose 8 μM). Animals were sacrificed at 6 weeks after treatment for tissue collection. Experimental animals were provided by Xiamen Fudexin Biotechnology Co., Ltd., Xiamen, Fujian, China.

### 2.12. Imaging Examination

Radiological assessment of IDD was performed using X-ray and MRI at week 8 post-puncture. X-ray images were acquired using a digital radiography system (United Imaging, Shanghai, China) under standardized conditions, and the disc height index (DHI%) was computed as the ratio of disc height to adjacent vertebral body height, as previously described [40]. MRI was performed on a 3.0 T magnetic resonance imaging (MRI) instrument (United Imaging, Shanghai, China) using a T2-weighted sequence (TR/TE = 3000/40 ms, FOV = 4 cm × 4 cm, slice thickness = 1 mm). Signal intensity within the nucleus pulposus on T2-weighted images was evaluated using the Pfirrmann grading system [41] by two independent, blinded observers. Pseudo-color MRI images were generated to visualize the signal intensity distribution.

### 2.13. Histological Examination

Harvested disc tissues were fixed in 10% neutral buffered formalin for 48 h, decalcified in 10% EDTA solution (pH 7.4) for 4 weeks at 4 °C, and then processed for paraffin embedding. Serial sections (5 µm) were cut and stained with hematoxylin and eosin (H&E) for morphological evaluation. Histological sections were assessed using a modified histological scoring system as previously reported [40]. The Pfirrmann-equivalent histological scores were assigned based on the cellularity of the nucleus pulposus, the integrity of the annulus fibrosus, and the boundary between the NP and AF. All histological evaluations were performed by at least two independent, blinded observers.

### 2.14. Statistical Analysis

Data are expressed as mean ± standard deviation (SD). Statistical analysis was carried out with GraphPad Prism 9.0 (GraphPad Software, San Diego, CA, USA). Comparisons between two groups relied on Student’s *t*-test, while differences across multiple groups were analyzed using one-way analysis of variance (ANOVA) followed by Tukey’s post hoc test. Statistical significance was defined as a *p*-value below 0.05.

## 3. Results and Discussion

### 3.1. Characterization of Nuc-LNPs

**Figure 1:**
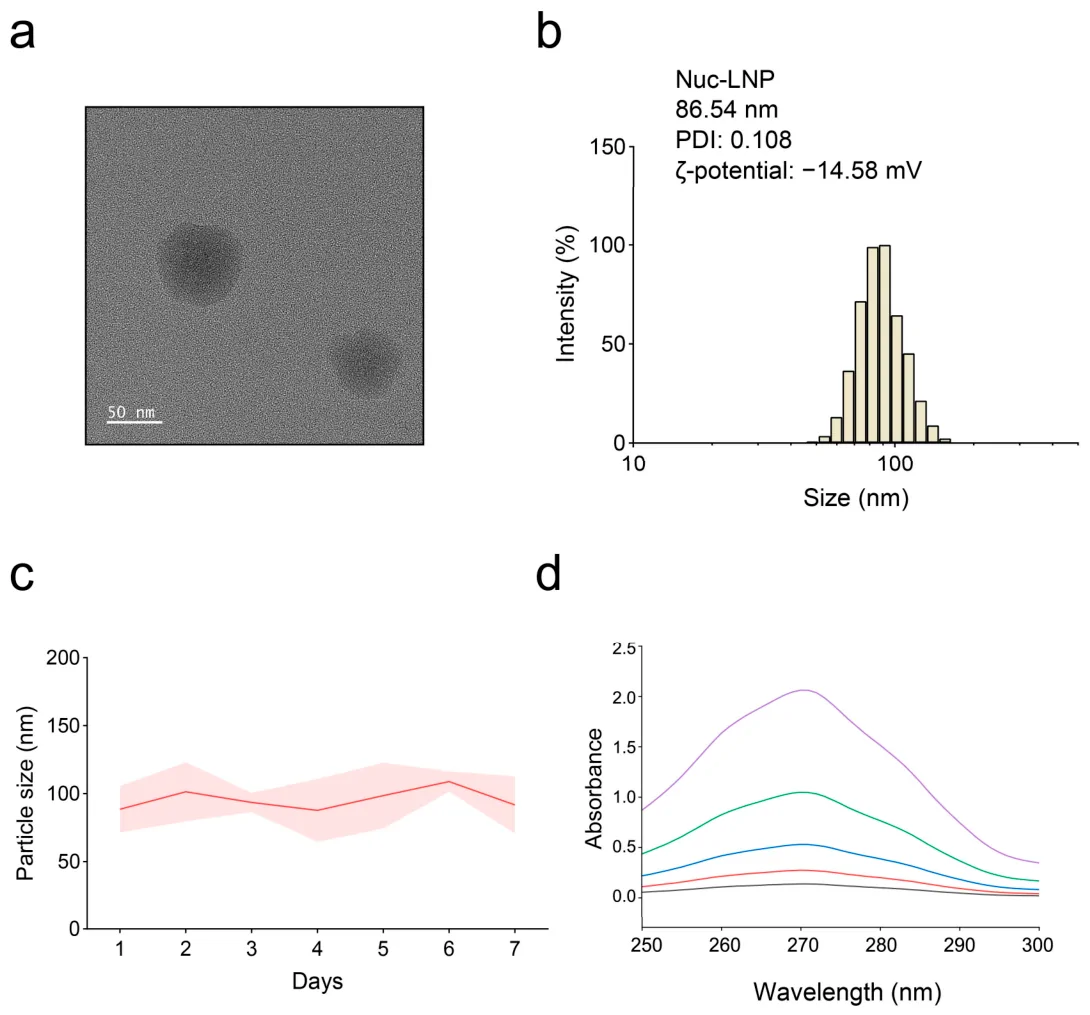
Preparation and characterization of nuciferine-loaded lipid nanoparticles (Nuc-LNPs). (**a**) Transmission electron microscopy (TEM) image of Nuc-LNPs, confirming their uniform spherical morphology. Scale bar: 50 nm. (**b**) Size distribution histogram of Nuc-LNPs measured by dynamic light scattering (DLS), showing a hydrodynamic diameter of 86.54 nm, PDI of 0.108, and zeta potential of −14.58 mV. (**c**) Storage stability of Nuc-LNPs assessed by particle size change over 7 days at 4 °C. (**d**) UV-Vis absorption spectra of Nuc at varying concentrations (3.9, 7.8, 15.6, 31.3, 62.5 μg/mL). The absorbance increased gradually with rising Nuc concentration, and the characteristic absorption peak at 270 nm was applied for quantitative analysis.

The molecular structure of Nuc is shown in Figure S1. Nuc-LNPs were fabricated via a microfluidic mixing technique, and their morphology was verified by transmission electron microscopy (TEM), which revealed a homogeneous population of spherical nanoparticles (Figure 1a). Dynamic light scattering (DLS) measurements indicated a mean hydrodynamic diameter of approximately 86.54 nm and a polydispersity index (PDI) of 0.108, reflecting a narrow size distribution with good uniformity (Figure 1b). To assess storage stability, particle size was tracked over a 7-day period at 4 °C, during which no appreciable size variation was detected (Figure 1c), confirming the robust colloidal stability of the Nuc-LNP formulation. The Nuc-LNPs exhibited a zeta potential of approximately −14.58 mV. Notably, this moderate negative surface potential is insufficient to sustain long-term colloidal stability merely through electrostatic repulsion, as reliable colloidal stabilization generally requires an absolute zeta potential value above 30 mV. The favorable colloidal stability of Nuc-LNPs may be primarily attributed to the steric stabilization effect derived from the DMG-PEG2000 component in the LNP formulation, which has been widely validated in conventional LNP systems. The UV absorption profile of nuciferine displayed a distinctive peak at 270 nm, which served as the analytical wavelength for quantitative determination. A linear standard curve spanning concentrations from 3.9 to 62.5 μg/mL was constructed with excellent linearity (R^2^ > 0.999) (Figure 1d). The resulting encapsulation efficiency (EE%) was determined to be 75.65%. FTIR reveals that characteristic peaks such as the (CH_2_)n stretch at 2930 cm^−1^ (LNP), the -CO- signal at 1728 cm^−1^ (LNP), the aromatic ring skeletal vibration at 1655 cm^−1^ (HA-PBA), and the aromatic ring skeletal vibrations at 1582 cm^−1^ and 1593 cm^−1^ (Nuc) can be all found in Nuc-LNP@MN, which collectively verified the complete integration of all components within the microneedle system (Figure S2).

### 3.2. Preparation, Physicochemical Properties and Characterization of Nuc-LNP@MN

**Figure 2:**
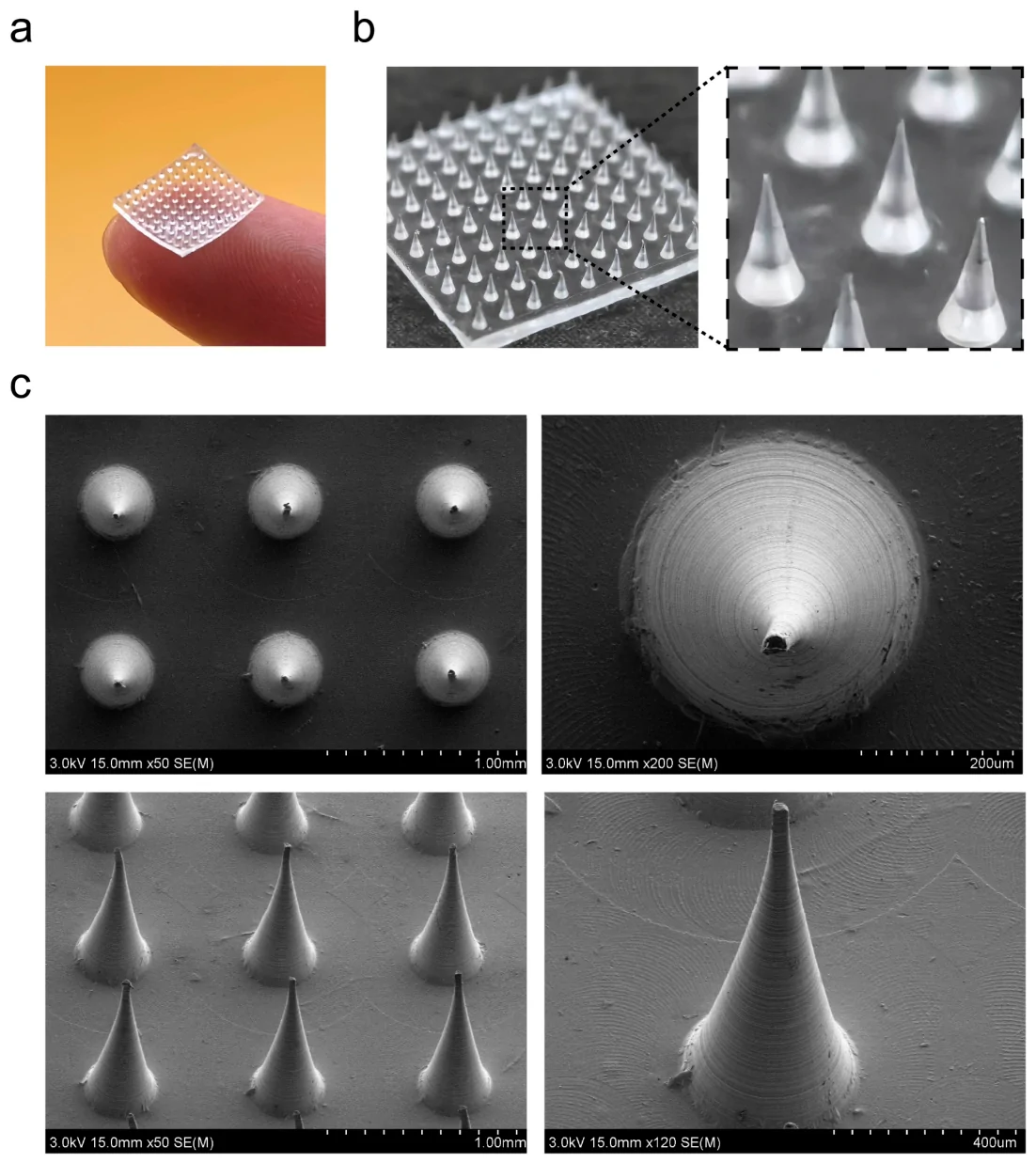
Macroscopic morphology and fabrication of the Nuc-LNP@MN patch. (**a**) Photograph of the Nuc-LNP@MN patch taken with a smartphone (Mate 60 Pro, Huawei, Shenzhen, Guangdong, China), showing the overall uniform patch morphology. (**b**) Close-up smartphone image of the microneedle array surface, demonstrating well-defined tip alignment. (**c**) Scanning electron microscopy (SEM) image of the microneedle array, confirming sharp pyramidal tip geometry with smooth surfaces. Scale bar as indicated.

The Nuc-LNP@MN arrays were successfully fabricated using a vacuum-assisted molding process. Macroscopic photographs captured by smartphone revealed a uniform microneedle patch with well-defined tip alignment (Figure 2a,b). SEM imaging confirmed that the microneedles exhibited a sharp, pyramidal tip geometry with smooth surfaces and uniform height (Figure 2c). The needle-to-needle spacing and tip sharpness were consistent across the entire array, demonstrating the reproducibility of the fabrication process.

**Figure 3:**
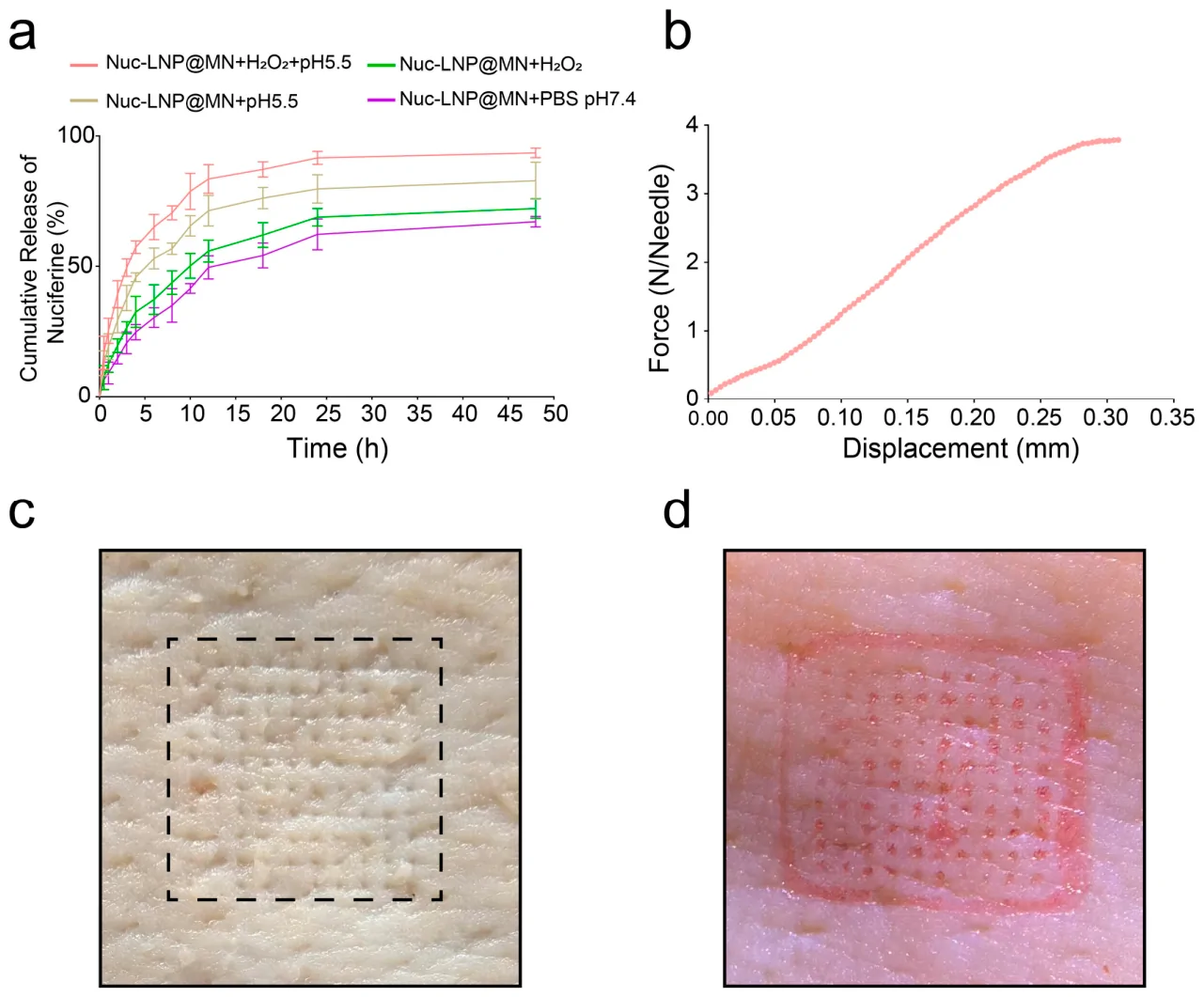
In vitro drug release behavior and mechanical characterization of Nuc-LNP@MN. (**a**) Cumulative drug release profiles of Nuc-LNP@MN under different stimulus conditions—PBS (control, pH 7.4), ROS (100 μM H_2_O_2_), acidic pH (pH 5.0), and dual stimuli (ROS + acidic pH)—demonstrating ROS/pH dual-responsive drug release. (**b**) Compression force–displacement curves of Nuc-LNP@MN arrays measured by universal testing machine, confirming sufficient mechanical strength for skin penetration. (**c**) Macroscopic photograph and (**d**) rhodamine B staining of porcine skin after Nuc-LNP@MN application, demonstrating successful skin penetration.

The stimuli-responsive behavior of the HA-PBA matrix originates from dynamic covalent boronic ester bonds formed between the PBA groups and the cis-diol moieties on neighboring HA chains [42]. Under physiological conditions (neutral pH, low ROS), these boronic ester bonds are stable and maintain the three-dimensional crosslinked network that gives the microneedle tips their solid, load-bearing structure. This crosslinking chemistry underpins both the structural integrity during fabrication and storage, and the stimuli-triggered disassembly during therapeutic application. Given that HA-PBA possesses inherent ROS/pH-responsive properties through its boronic ester bonds, the stimulus-triggered release behavior of Nuc-LNP@MN was first characterized. As shown in Figure 3a, all four groups exhibited a biphasic release pattern characterized by a rapid burst phase within the first 12 h followed by a gradual plateau. Under dual-stimulus conditions (H_2_O_2_ + pH 5.5), which most closely recapitulate the oxidative and acidic microenvironment of degenerated IVDs, cumulative release reached 83.6 ± 5.5% at 12 h and 93.5 ± 1.9% at 48 h—the highest among all groups. Under acidic pH alone (pH 5.5), release reached 71.4 ± 5.9% at 12 h and 82.9 ± 7.1% at 48 h, while under ROS alone (H_2_O_2_), release reached 55.9 ± 4.2% at 12 h and 72.2 ± 3.8% at 48 h. In the physiological control (PBS, pH 7.4), mimicking the healthy IVD microenvironment, the lowest release was observed (49.6 ± 4.5% at 12 h; 67.1 ± 2.1% at 48 h). Compared with the physiological control, the dual-stimulus condition increased 48 h cumulative release by 26.4 percentage points, the acidic pH condition by 15.8 percentage points, and the ROS condition by 5.1 percentage points. Notably, the pH 5.5 condition alone (82.9%) outperformed H_2_O_2_ alone (72.2%) by 10.7 percentage points at 48 h, indicating that acidic pH is the dominant single stimulus driving drug release from this system, while ROS provides a supplementary contribution; their combination produces a synergistic enhancement that maximizes payload liberation in the degenerated disc microenvironment. These results confirm that the ROS- and pH-rich microenvironment of the degenerated disc can effectively trigger on-demand drug release from the Nuc-LNP@MN system. The degradation kinetics of the HA-PBA matrix under these conditions follow a stimulus-intensity-dependent pattern: the release rate under dual stimuli (ROS + acidic pH) was markedly faster than under either stimulus alone, indicating synergistic acceleration of boronic ester hydrolysis. Mechanistically, under elevated ROS, hydrogen peroxide oxidizes the trigonal planar boronic acid to a tetrahedral boronate anion intermediate that is susceptible to hydrolysis, thereby cleaving the crosslinks. Under acidic pH, protonation of the PBA moieties shifts the boronate ester equilibrium toward the open (hydrolyzed) form [43]. In the pathological IDD microenvironment, ROS levels are significantly elevated due to mitochondrial dysfunction and inflammatory infiltration, while interstitial pH drops to approximately 5.0–6.5 as a result of anaerobic glycolysis and lactic acid accumulation. The Nuc-LNP@MN system is therefore engineered to remain intact under physiological conditions, while undergoing selective triggered disassembly within the degenerative disc microenvironment, achieving precision on-demand drug delivery.

The mechanical performance of the microneedle array is critical for successful skin penetration. Compression testing using the universal testing machine demonstrated that the Nuc-LNP@MN patches exhibited sufficient mechanical strength to withstand the forces required for skin insertion (Figure 3b). The force–displacement curves showed that the microneedles maintained structural integrity under compression forces well above the reported threshold for skin penetration (~0.1 N/needle), confirming adequate stiffness for practical application. To further verify the skin penetration capability, Nuc-LNP@MN patches were applied to freshly excised porcine skin and the resulting puncture holes were visualized by trypan blue staining. As shown in Figure 3c,d, a regular pattern of rhodamine B-stained holes corresponding to the microneedle array arrangement was clearly observed; all needle tips successfully created discrete micropores matching the array geometry. The puncture holes displayed uniform depth and diameter distribution across the patch, confirming that the microneedles successfully penetrated the skin barrier in an ex vivo model. These results collectively demonstrate that the HA-PBA crosslinked matrix provides sufficient mechanical rigidity for reliable transdermal penetration, while the dynamic boronic ester bonds endow the system with the capacity for microenvironment-triggered degradation and drug release upon insertion into the disc-adjacent tissue.

### 3.3. Cytocompatibility of Nuc-LNP@MN

**Figure 4:**
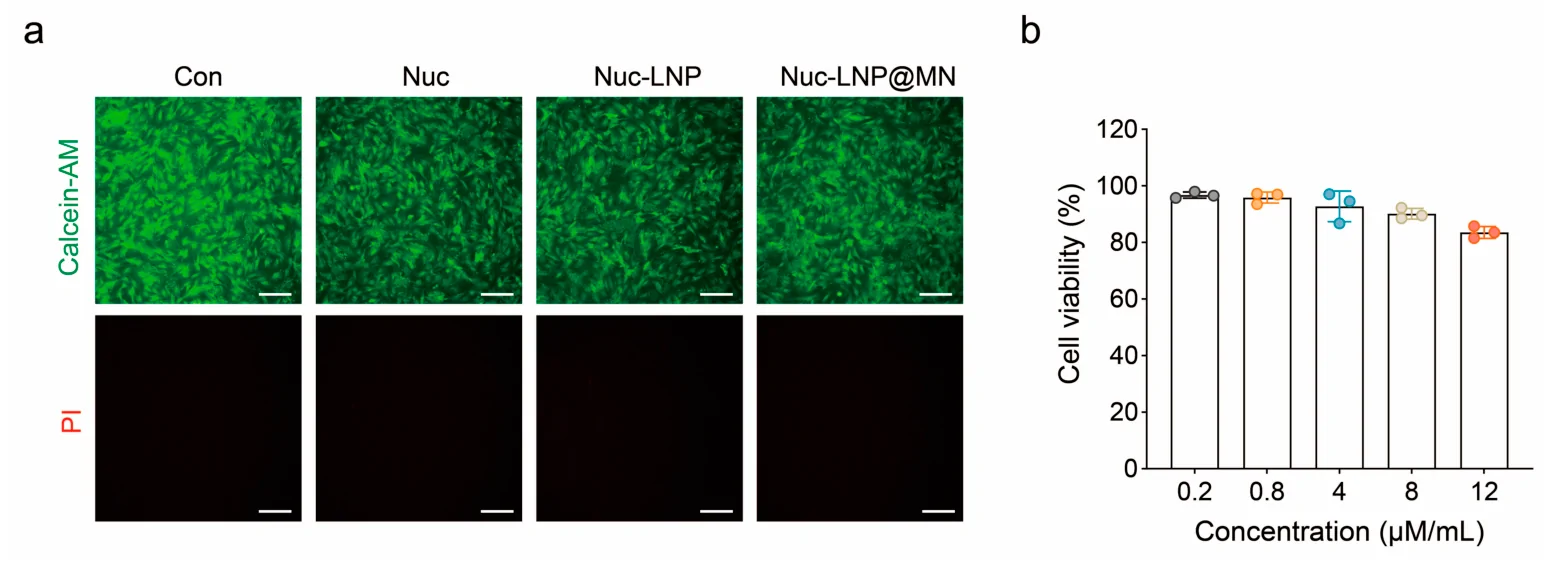
Cytocompatibility assessment of Nuc-LNP@MN in NPCs. (**a**) Representative fluorescence images of Calcein-AM/PI live/dead staining in NPCs co-cultured with Con, Nuc, Nuc-LNP, and Nuc-LNP@MN groups via a Transwell system. Green: live cells (Calcein-AM); red: dead cells (PI). Scale bar: 200 μm. (**b**) Cell viability of NPCs treated with Nuc-LNPs at concentrations of 0.2, 0.8, 4, 8, and 12 μM (nuciferine equivalent) as measured by CCK-8 assay. Data are presented as mean ± SD (*n* = 3).

The cytocompatibility of the Nuc-LNP@MN system was systematically evaluated. Calcein-AM/PI live/dead staining showed that cells treated with Nuc-LNPs in the Transwell co-culture system displayed predominantly green (live) fluorescence with minimal red (dead) signal, comparable to the untreated control (Figure 4a), indicating no apparent cytotoxic effect across all tested groups. CCK-8 assay results further demonstrated that treatment with Nuc-LNPs at concentrations ranging from 0.2 to 12 μM (nuciferine equivalent) did not significantly reduce the viability of NPCs compared to the control group (Figure 4b), with viability exceeding 90% across all tested concentrations. These results demonstrate the excellent biocompatibility of Nuc-LNPs and the Nuc-LNP@MN system for NPCs.

### 3.4. Anti-Inflammatory and ECM-Protective Effects of Nuc-LNP@MN In Vitro

**Figure 5:**
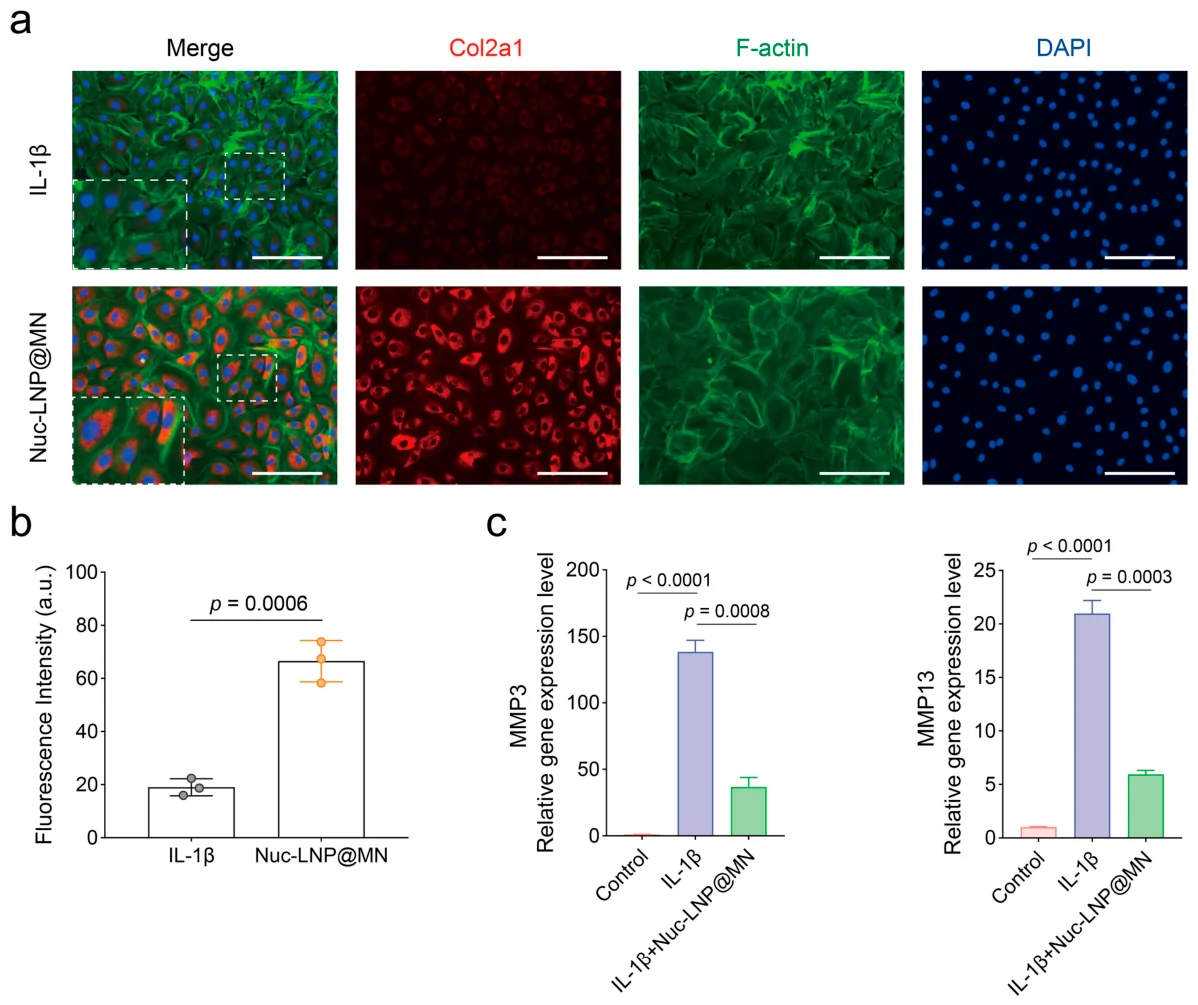
Anti-inflammatory and extracellular matrix (ECM)-protective effects of Nuc-LNP@MN in IL-1β-stimulated NPCs. (**a**) Representative immunofluorescence images showing Col2a1 (red), F-actin (green), and DAPI (blue) staining in IL-1β-stimulated NPCs treated with Nuc-LNP@MN. Scale bar: 200 µm. (**b**) Quantification of Col2a1 fluorescence intensity. (**c**) Quantitative RT-PCR analysis of MMP3 (**left**) and MMP13 (**right**) mRNA expression levels in each group. Data are expressed as mean ± SD. *p* < 0.05 denotes statistical significance.

To assess the therapeutic performance of Nuc-LNP@MN under IDD-mimicking conditions, NPCs were first challenged with IL-1β (10 ng/mL) and subsequently subjected to the following treatment regimens: untreated control (Con), IL-1β alone and Nuc-LNP@MN. Immunofluorescence analysis of Col2a1, a principal ECM protein synthesized by NPCs, demonstrated a marked decline in Col2a1 levels within the IL-1β-treated group relative to the control, consistent with inflammation-driven ECM breakdown. Remarkably, Nuc-LNP@MN administration led to substantial recovery of Col2a1 expression, as reflected by enhanced red fluorescence signal (Figure 5a). Quantitative evaluation verified that Nuc-LNP@MN significantly elevated Col2a1 fluorescence intensity relative to the IL-1β group (*p* < 0.001) (Figure 5a, right panel).

RT-PCR quantification of MMP3 and MMP13, two pivotal matrix metalloproteinases implicated in ECM breakdown during IDD, showed that IL-1β exposure substantially elevated the transcript levels of both enzymes. Treatment with Nuc-LNP@MN markedly attenuated MMP3 and MMP13 mRNA expression relative to the IL-1β group (*p* < 0.001), while free nuciferine exhibited comparatively weaker inhibitory effects, suggesting that the LNP encapsulation combined with the microneedle delivery platform conferred a synergistic therapeutic advantage (Figure 5b). Collectively, these findings indicate that Nuc-LNP@MN confers effective protection against IL-1β-mediated inflammatory ECM destruction in NPCs.

### 3.5. Mitophagy Activation by Nuc-LNP@MN

**Figure 6:**
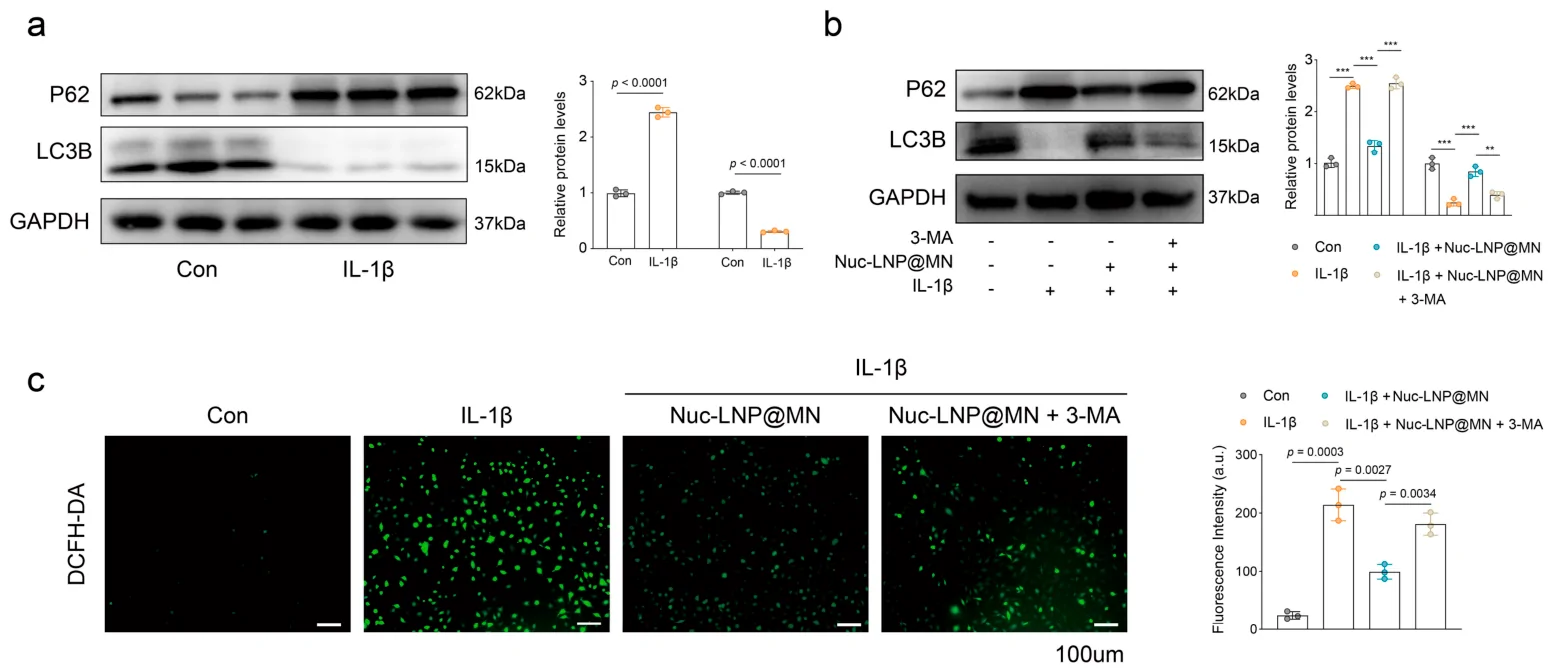
Nuc-LNP@MN activates mitophagy and reduces intracellular ROS in IL-1β-stimulated NPCs. (**a**) Western blot analysis of P62 and LC3B protein expression in Con and IL-1β-treated NPCs, with GAPDH as loading control. Right panel: quantification of band intensities normalized to GAPDH. (**b**) Western blot analysis of P62 and LC3B in IL-1β-treated NPCs across groups, with corresponding quantification, confirming autophagy-dependent therapeutic effects. (**c**) Representative fluorescence images of DCFH-DA-stained NPCs showing intracellular ROS levels in Con, IL-1β, Nuc-LNP@MN, and Nuc-LNP@MN + 3-MA groups. Right panel: quantification of mean DCF fluorescence intensity. Scale bar: 100 μm. Data are presented as mean ± SD. ** *p* < 0.01; *** *p* < 0.001.

To elucidate the mechanism by which Nuc-LNP@MN exerts its therapeutic effects, we examined mitophagy-related protein expression. Western blot analysis showed that IL-1β stimulation decreased LC3B-II expression and increased P62 accumulation in NPCs, consistent with impaired autophagic flux. In contrast, Nuc-LNP@MN treatment significantly upregulated LC3B-II and downregulated P62 compared to the IL-1β group, indicating restoration of mitophagy (Figure 6a). To confirm that the anti-inflammatory and ECM-protective effects of Nuc-LNP@MN were mediated by autophagy, cells were co-treated with the autophagy inhibitor 3-MA. The addition of 3-MA abrogated the Nuc-LNP@MN-induced changes in LC3B-II and P62 expression (Figure 6b), confirming the autophagy-dependent mechanism (Figure S3). Consistently, immunoblotting showed that IL-1β suppressed the expression of mitophagy-related PINK1 and Parkin, and Nuc-LNP@MN treatment reversed this inhibition, activating PINK1/Parkin-dependent mitophagy (Figure S3). Furthermore, intracellular ROS levels, as measured by DCFH-DA fluorescence, were markedly elevated in IL-1β-stimulated NPCs and significantly reduced following Nuc-LNP@MN treatment, reflecting the capacity of restored mitophagy to clear dysfunctional mitochondria and suppress mitochondrial ROS generation (Figure 6c). Co-treatment with 3-MA partially reversed this ROS-scavenging effect, further confirming that the suppression of intracellular ROS by Nuc-LNP@MN is mediated through mitophagy activation. The provided Western blot results clearly demonstrate that IL-1β challenge disrupts ECM balance by elevating matrix-degrading Adamts5 and MMP13 while downregulating Col2a1. Nuc-LNP@MN treatment robustly rescues ECM anabolic–catabolic imbalance and mitigates inflammatory damage, superior to free Nuc and blank Nuc-LNPs (Figure S4a). IL-1β breaks the anabolic–catabolic balance in NPCs: it increases catabolic enzymes MMP13 and ADAMTS5 and reduces anabolic Col2a1. By contrast, Nuc-LNP@MN effectively reverses this pathological shift, restoring Col2a1 while downregulating MMP13/ADAMTS5 (Figure S4b). Activated mitophagy scavenges mitochondrial ROS, inhibits NF-κB-driven inflammatory cytokine production, suppresses Bax/Bcl-2 apoptotic cascades, and alleviates cellular senescence as well as SASP, thereby restoring the physiological balance of extracellular matrix (ECM) metabolism in nucleus pulposus cells. These results strongly suggest that Nuc-LNP@MN exerts its cytoprotective effects primarily through activation of mitophagy, which in turn clears damaged mitochondria and reduces intracellular ROS production.

### 3.6. In Vivo Therapeutic Efficacy of Nuc-LNP@MN

**Figure 7:**
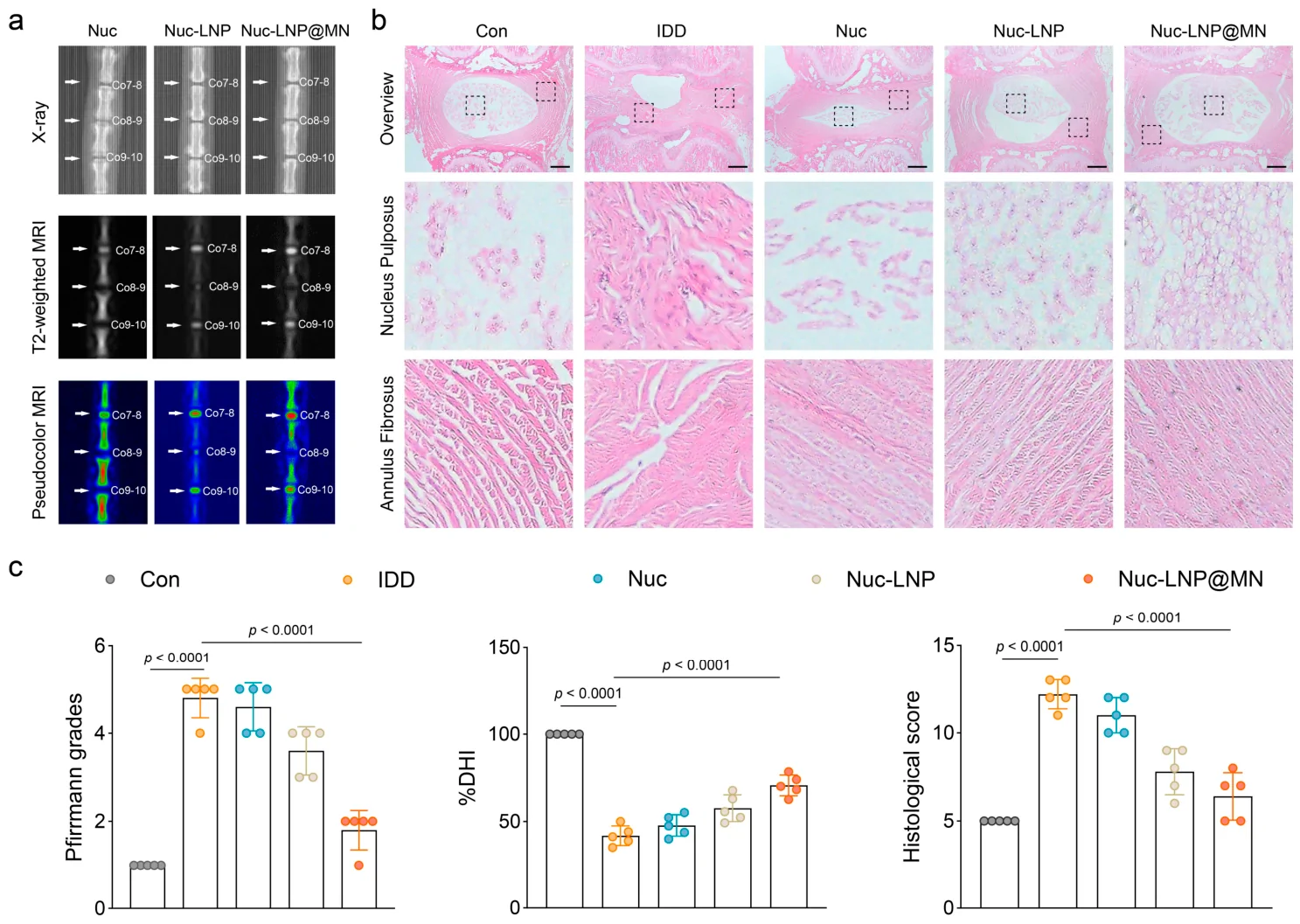
In vivo therapeutic efficacy of Nuc-LNP@MN in a rat needle-puncture IDD model. (**a**) Representative lateral X-ray images (**top**), T2-weighted MRI (**middle**), and pseudo-color MRI images (**bottom**) of coccygeal discs in Con, IDD, Nuc, Nuc-LNP, and Nuc-LNP@MN groups. Arrows indicate the targeted disc levels. (**b**) Representative H&E staining of nucleus pulposus (**upper**) and annulus fibrosus (**lower**) sections in each group. Scale bar: 500 μm. (**c**) Statistical analysis of Pfirrmann grades (MRI, **left**), disc height index (%DHI, **middle**), and histological scores (**right**) among all groups. Data are presented as mean ± SD.

The in vivo therapeutic efficacy of Nuc-LNP@MN was evaluated in a rat needle-puncture IDD model. Radiological assessment by X-ray and MRI was performed to monitor disc degeneration over time. X-ray images showed that the IDD group exhibited significant disc space narrowing compared to the sham control (Con) group, with a markedly reduced disc height index (DHI%). Treatment with Nuc-LNP@MN substantially preserved disc height, and the DHI% was significantly higher than that of the IDD group (*p* < 0.0001). T2-weighted MRI demonstrated that the IDD group showed significantly reduced signal intensity in the nucleus pulposus region, indicative of water content loss and disc degeneration, while the Nuc-LNP@MN group maintained a relatively high and uniform T2 signal intensity, approaching that of the healthy Con group (Figure 7a). Pfirrmann grading scores for MRI were significantly lower (better) in the Nuc-LNP@MN group compared to the IDD, Nuc, and Nuc-LNP groups (*p* < 0.0001) (Figure 7c).

Histological examination with H&E staining further confirmed the therapeutic efficacy of Nuc-LNP@MN. The IDD group showed characteristic histopathological features of disc degeneration, including disorganized and fibrotic nucleus pulposus, disrupted annulus fibrosus lamellae, and reduced cellularity. In contrast, the Nuc-LNP@MN group exhibited significantly better-preserved disc architecture, with a more abundant and gelatinous nucleus pulposus and more organized annulus fibrosus structure (Figure 7b). Histological scores were significantly lower in the Nuc-LNP@MN group compared to all other treatment groups (*p* < 0.0001) (Figure 7c). In vivo ELISA measurements further verified that the expression of pro-inflammatory cytokines TNF-α and IL-6 was greatly increased in the IDD group, while Nuc-LNP@MN administration substantially suppressed their accumulation (Figure S5). Taken together, these in vivo results demonstrate that Nuc-LNP@MN effectively retards disc degeneration and preserves disc structure and function in a rat IDD model, supporting its translational potential.

### 3.7. Limitations and Future Perspectives

Despite the encouraging findings regarding the Nuc-LNP@MN system, several limitations must be acknowledged. The acute needle-puncture model induces rapid mechanical disruption, which fails to faithfully recapitulate the chronic, multifactorial pathogenesis of clinical IDD. Because human IDD progresses over years due to cumulative mechanical stress, age-related cellular senescence, and gradual nutritional deprivation, this acute injury may lead to an overestimation of therapeutic efficacy. Future investigations should incorporate naturally aged models or sustained mechanical overloading systems that better simulate the human biomechanical environment. Furthermore, the brief 6-week observation period is insufficient to comprehensively assess long-term safety and durability. It remains unclear whether restored mitophagic activity and attenuated ECM degradation can be sustained indefinitely or if repeated administrations are necessary, warranting extended follow-up studies spanning 12 to 24 weeks.

This study was conducted exclusively in a rat model, whose coccygeal disc dimensions, biomechanical loading, and nutritional supply differ considerably from those of human lumbar discs. Crucially, the small disc size in rats may artificially facilitate drug penetration from the microneedle surface—an anatomical advantage that is unlikely to translate directly to larger human discs. To bridge this gap, future validation must be performed in large animal models, such as goats or sheep. Because their disc anatomy and degenerative characteristics more closely approximate human conditions, these models are essential to rigorously evaluate microneedle penetration efficiency, spatial drug distribution, and dose–response relationships at a clinically relevant scale before advancing toward clinical translation.

Looking ahead, several promising directions merit exploration to further advance this platform. Artificial intelligence (AI)-assisted algorithms could be leveraged to optimize biomaterial compositions, predict stimulus-responsive drug release kinetics, and accelerate the rational design of next-generation microneedle formulations. Patient-specific, 3D-printed spinal implants tailored to individual disc geometry and degeneration severity could complement the microneedle delivery approach, enabling truly personalized spinal therapeutics [44]. Moreover, image-guided delivery systems—for example, ultrasound- or MRI-guided microneedle application—would allow real-time confirmation of drug deposition at the target disc level, improving precision and reducing variability [45]. Combination strategies integrating Nuc-LNP@MN with regenerative medicine approaches, such as co-delivery of growth factors (e.g., TGF-β1, GDF-5) or incorporation of nucleus pulposus-derived stem cell therapies, hold particular promise for achieving synergistic disc repair that simultaneously addresses both the inflammatory microenvironment and tissue regeneration.

## 4. Conclusions

This study developed a novel, minimally invasive, and stimulus-responsive nuciferine-loaded lipid nanoparticle microneedle system (Nuc-LNP@MN) for IDD treatment. The fabricated Nuc-LNPs displayed uniform spherical morphology, with a particle size of 86.54 nm, an encapsulation efficiency of 75.65%, and satisfactory colloidal stability. This platform combines ROS/pH dual-responsive HA-PBA microneedles with nuciferine-encapsulated LNPs, enabling precise, on-demand drug release in response to the acidic and oxidative pathological microenvironment of degenerated discs. In vitro experiments confirmed the excellent biocompatibility of Nuc-LNP@MN, maintaining over 90% viability of NPCs. Mechanistically, Nuc-LNP@MN activated mitophagy by upregulating LC3B-II and downregulating P62, which disrupted the oxidative stress–inflammation vicious cycle. It also efficiently prevented ECM degradation by downregulating the catabolic factors MMP3 and MMP13. Corroborative in vitro and in vivo results validated that Nuc-LNP@MN significantly improved key disc repair indexes, including an elevated disc height index (DHI%), improved Pfirrmann grade, and restored histological scores (*p* < 0.05), thereby preserving disc structure and ameliorating IDD progression. Overall, this study verifies nuciferine as an effective mitophagy activator for IDD therapy and proves the promising application of stimulus-responsive microneedle-based nanosystems in localized, minimally invasive treatment of intervertebral disc diseases.

## Figures and Tables

**Table 1:** Primer sequences used for quantitative RT-PCR.

Gene Name	Forward (5′-3′)	Reverse (5′-3′)
*Rat-MMP3*	GCTCATCCTACCCATTGCAT	GCTTCCCTGTCATCTTCAGC
*Rat-MMP13*	ACCATCCTGTGACTCTTGCG	TTCACCCACATCAGGCACTC
*Rat-GAPDH*	GGTGAAGGTCGGTGTGAACG	CTCGCTCCTGGAAGATGGTG

## Data Availability

The data are available from the corresponding author upon reasonable request.

## Supplementary Materials

The following supporting information can be downloaded at https://www.mdpi.com/article/10.3390/pharmaceutics18080997/s1. Figure S1: Chemical molecular structure of nuciferine; Figure S2. Fourier-transform infrared (FTIR) spectra of HA-PBA, free Nuc, blank LNP, Nuc-LNP and Nuc-LNP@MN; Figure S3. Nuc-LNP@MN activates PINK1/Parkin-mediated mitophagy in IL-1β-treated nucleus pulposus cells; Figure S4. Nuc-LNP@MN alleviates IL-1β-triggered extracellular matrix degradation in NPCs. (**a**) MMP13 and Col2a1 in control, IL-1β, and IL-1β + Nuc-LNP@MN groups. (**b**) Adamts5, MMP13 and Col2a1 expression among free Nuc, blank Nuc-LNP and Nuc-LNP@MN treatment groups upon IL-1β stimulation; Figure S5. ELISA results showing tissue concentrations of TNF-α and IL-6 across Con, IDD, and IDD + Nuc-LNP@MN groups. Data are presented as individual points with mean ± SD. *p* values were obtained from one-way ANOVA. *p* < 0.05 indicated statistically significance (n = 5).
