# Supplementary material for: Nuciferine-Loaded Lipid Nanoparticle Microneedles Alleviate Intervertebral Disc Degeneration by Restoring Nucleus Pulposus Cell Homeostasis
Source: Pharmaceutics. 2026 Aug 13;18(8):997. doi: 10.3390/pharmaceutics18080997 (PMC13516712; doi:10.3390/pharmaceutics18080997)
Supplement: Supplementary file 1 [file pharmaceutics-18-00997-s001.zip › pharmaceutics-4421405-supplementary.pdf]

Nuciferine-Loaded Lipid Nanoparticle Microneedles Alleviate Intervertebral Disc Degeneration by Restoring Nucleus Pulposus Cell Homeostasis

Yifan Ding [1,#], Genchun Wang [2,#], Yucheng Wang [1], Songwei Tan [3,*], Yixin Cai [1,*]

[1] Department of Thoracic Surgery, Tongji Hospital, Tongji Medical College, Huazhong University of Science and Technology, Wuhan 430030, China.

[2] Department of Joint Surgery, Orthopedic Medical Center, Fujian Medical University Union Hospital, Fuzhou 350001, China.

[3] School of Pharmacy, Tongji Medical College, Huazhong University of Science and Technology, Wuhan 430030, China.

# These authors contributed equally to this work

* Correspondence:

Yixin Cai (caiyixin@tjh.tjmu.edu.cn);

Songwei Tan (tansongwei@gmail.com; tansw@hust.edu.cn)

Running title: Nuciferine Microneedle System for Intervertebral Disc Degeneration

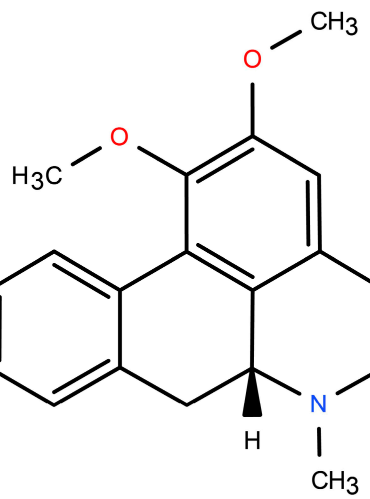

Figure S1. Chemical molecular structure of nuciferine.

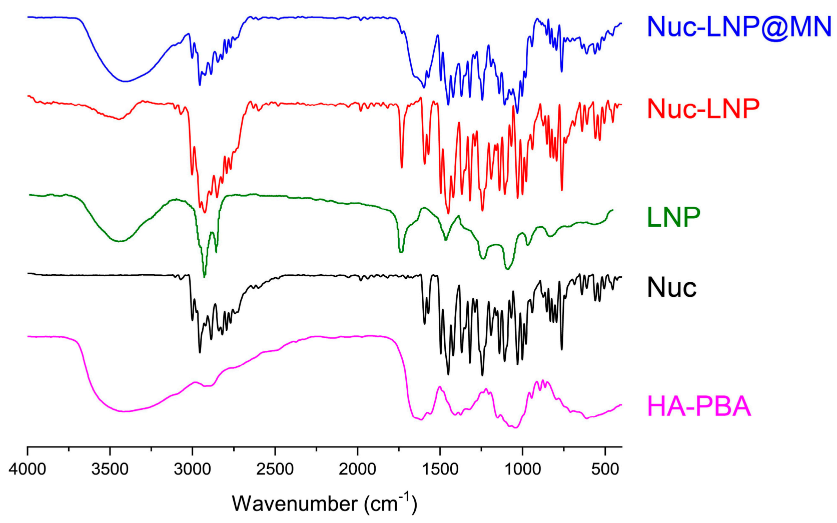

Figure S2. Fourier-transform infrared (FTIR) spectra of HA-PBA, free Nuc, blank LNP, Nuc-LNP and Nuc-LNP@MN

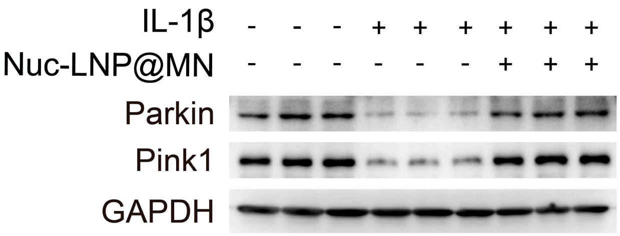

Figure S3. Nuc-LNP@MN activates PINK1/Parkin-mediated mitophagy in IL-1β-treated nucleus pulposus cells.

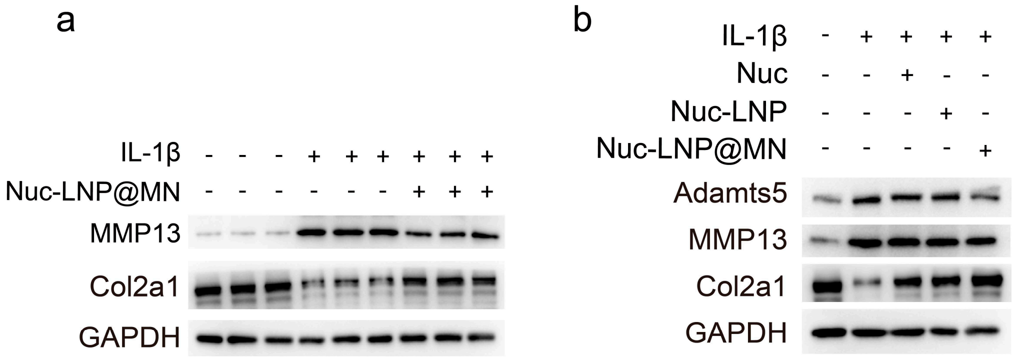

Figure S4. Nuc-LNP@MN alleviates IL-1β-triggered extracellular matrix degradation in NPCs. (a) MMP13 and Col2a1 in control, IL-1β, and IL-1β + Nuc-LNP@MN groups. (b) Adamts5, MMP13 and Col2a1 expression among free Nuc, blank Nuc-LNP and Nuc-LNP@MN treatment groups upon IL-1β stimulation.

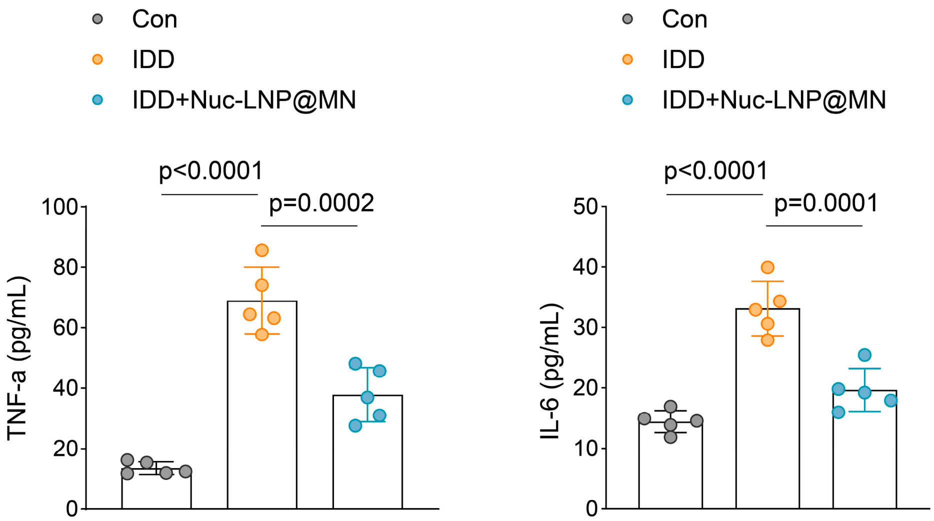

Figure S5. ELISA results showing tissue concentrations of TNF-α and IL-6 across Con, IDD, and IDD + Nuc-LNP@MN groups. Data are presented as individual points with mean ± SD. P values were obtained from one-way ANOVA. P<0.05 indicated

statistically significance (n=5).
